# libgapmis: extending short-read alignments

**DOI:** 10.1186/1471-2105-14-S11-S4

**Published:** 2013-11-04

**Authors:** Nikolaos Alachiotis, Simon Berger, Tomáš Flouri, Solon P Pissis, Alexandros Stamatakis

**Affiliations:** 1 Heidelberg Institute for Theoretical Studies, Heidelberg, Germany; 2 Florida Museum of Natural History, University of Florida, Gainesville, FL, USA

## Abstract

**Background:**

A wide variety of short-read alignment programmes have been published recently to tackle the problem of mapping millions of short reads to a reference genome, focusing on different aspects of the procedure such as time and memory efficiency, sensitivity, and accuracy. These tools allow for a small number of mismatches in the alignment; however, their ability to allow for gaps varies greatly, with many performing poorly or not allowing them at all. The *seed-and-extend *strategy is applied in most short-read alignment programmes. After aligning a substring of the reference sequence against the high-quality prefix of a short read--*the seed*--an important problem is to find the best possible alignment between a substring of the reference sequence succeeding and the remaining suffix of low quality of the read--*extend*. The fact that the reads are rather short and that the gap occurrence frequency observed in various studies is rather low suggest that aligning (parts of) those reads with a single gap is in fact desirable.

**Results:**

In this article, we present libgapmis, a library for extending pairwise short-read alignments. Apart from the standard CPU version, it includes ultrafast SSE- and GPU-based implementations. libgapmis is based on an algorithm computing a modified version of the traditional dynamic-programming matrix for sequence alignment. Extensive experimental results demonstrate that the functions of the CPU version provided in this library accelerate the computations by a factor of 20 compared to other programmes. The analogous SSE- and GPU-based implementations accelerate the computations by a factor of 6 and 11, respectively, compared to the CPU version. The library also provides the user the flexibility to split the read into fragments, based on the observed gap occurrence frequency and the length of the read, thereby allowing for a variable, but bounded, number of gaps in the alignment.

**Conclusions:**

We present libgapmis, a library for extending pairwise short-read alignments. We show that libgapmis is better-suited and more efficient than existing algorithms for this task. The importance of our contribution is underlined by the fact that the provided functions may be seamlessly integrated into any short-read alignment pipeline. The open-source code of libgapmis is available at http://www.exelixis-lab.org/gapmis.

## Background

The problem of finding substrings of a text similar to a given pattern has been intensively studied over the past decades, and it is a central problem in a wide range of applications, including signal processing [1], information retrieval [2], searching for similarities among biological sequences [3], file comparison [4], spelling correction [5], and music analysis [6]. Some examples are recovering the original signals after their transmission over noisy channels, finding DNA subsequences after possible mutations, and text searching where there are typing or spelling errors.

Approximate string matching, in general, consists in locating all the occurrences of substrings inside a text *t *that are similar to a pattern *x*. It consists of producing the positions of the substrings of *t *that are at distance at most *k *from *x*, for a given natural number *k*. For the rest of this article, we assume that *k <*|*x*| ≤ |*t*|. We focus on online searching--the text cannot be preprocessed to build an index on it. There exist four main approaches to online approximate string matching: algorithms based on dynamic programming; algorithms based on automata; algorithms based on word-level parallelism; and algorithms based on filtering. We focus on algorithms based on dynamic programming. There mainly exist two different distances for measuring the approximation: the *edit distance *and the *Hamming distance*.

The edit distance between two strings, not necessarily of the same length, is the minimum cost of a sequence of elementary edit operations between these two strings. A restricted notion of this distance is obtained by considering the minimum number of edit operations rather than the sum of their costs. The Hamming distance between two strings of the same length is the number of positions where mismatches occur between the two strings.

Alignments are a commonly used technique to compare strings and are based on notions of distance [1] or of similarity between strings; for example, similarities among biological sequences [3]. Alignments are often computed by dynamic programming [2].

A *gap *is a sequence of consecutive insertions or deletions (indels) of letters in an alignment. The extensive use of alignments on biological sequences has shown that it can be desirable to penalise the formation of long gaps rather than penalising individual insertions or deletions of letters.

The notion of gap in a biological sequence can be described as the absence (respectively, presence) of a fragment, which is (respectively, is not) present in another sequence [7]. Gaps occur naturally in biological sequences as part of the diversity between individuals. In many biological applications, a single mutational event can cause the insertion (or deletion) of a large DNA fragment, so the notion of gap in an alignment is an important one. Moreover, the creation of gaps can occur in a wide, but bounded, range of sizes with almost equal likelihood.

A number of natural processes can cause gaps in DNA sequences: long pieces of DNA can be copied and inserted by a single mutational event; slippage during the replication of DNA may cause the same area to be repeated multiple times as the replication machinery loses its place on the template; an insertion in one sequence paired with a reciprocal deletion in one other may be caused by unequal cross-over in meiosis; insertion of transposable elements--jumping genes--into a DNA sequence; insertion of DNA by retroviruses; and translocations of DNA between chromosomes [8]. The accurate identification of gaps is shown to be fundamental in various studies on disorders; for example, on Hajdu-Cheney syndrome [9], a disorder of severe and progressive bone loss.

The focus of this work is directly motivated by the well-known and challenging application of *re-sequencing*--the assembly of a genome directed by a reference sequence. New developments in sequencing technologies (see [10-12], for example) allow whole-genome sequencing to be turned into a routine procedure, creating sequencing data in massive amounts. Short sequences (reads) are produced in huge amounts (tens of gigabytes), and in order to determine the part of the genome from which a read was derived, it must be mapped (aligned) back to some reference sequence, a few gigabases long.

A wide variety of short-read alignment programmes (e.g. Bowtie [13], SOAP2 [14], REAL [15], BWA [16], Bowtie2 [17]) were published in the past five years to address the challenge of efficiently mapping tens of millions of short reads to a genome, focusing on different aspects of the procedure: speed, sensitivity, and accuracy. These tools allow for a small number of mismatches in the alignment; however, their ability to allow for gaps varies greatly, with many performing poorly and other not allowing them at all.

**Figure 1:**
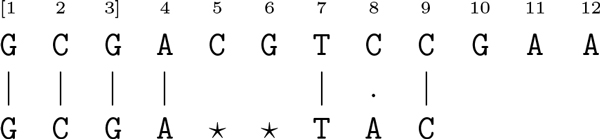
**Seed-and-extend strategy**. The alignment between the fragment of the reference sequence, starting at position 1 and ending at position 9, and the read with one mismatch at position 8 and a gap of length two inserted in the read after position 4. This figure was taken from [21].

Most short-read alignment programmes apply the well-known scheme of *seed-and-extend *[18]. After aligning a substring of the reference sequence against the *seed *(short high-quality prefix of the read - positions 1-3 in square brackets in Figure 1) very fast, a short-read alignment programme must compute the best possible alignment between a substring of the reference sequence succeeding and the remaining suffix of the read (low-quality suffix of the read - positions 4-9). This is achieved by allowing a bounded number of mismatches (position 8) and gaps (positions 5-6).

From Figure 1, we observe that a gap might need to be inserted in the leftmost position of the alignment (position 4). However, we are not able to know the length of the substring of the reference sequence to be aligned beforehand. Due to this observation, it is clear we need an intermediate between the global (Needleman-Wunsch algorithm [19], for example) and the local alignment (Smith-Waterman algorithm [20], for example), known as *semi-global *alignment, that allows the insertion of a gap at the end of an alignment with no penalty (positions 10-12).

**Figure 2:**
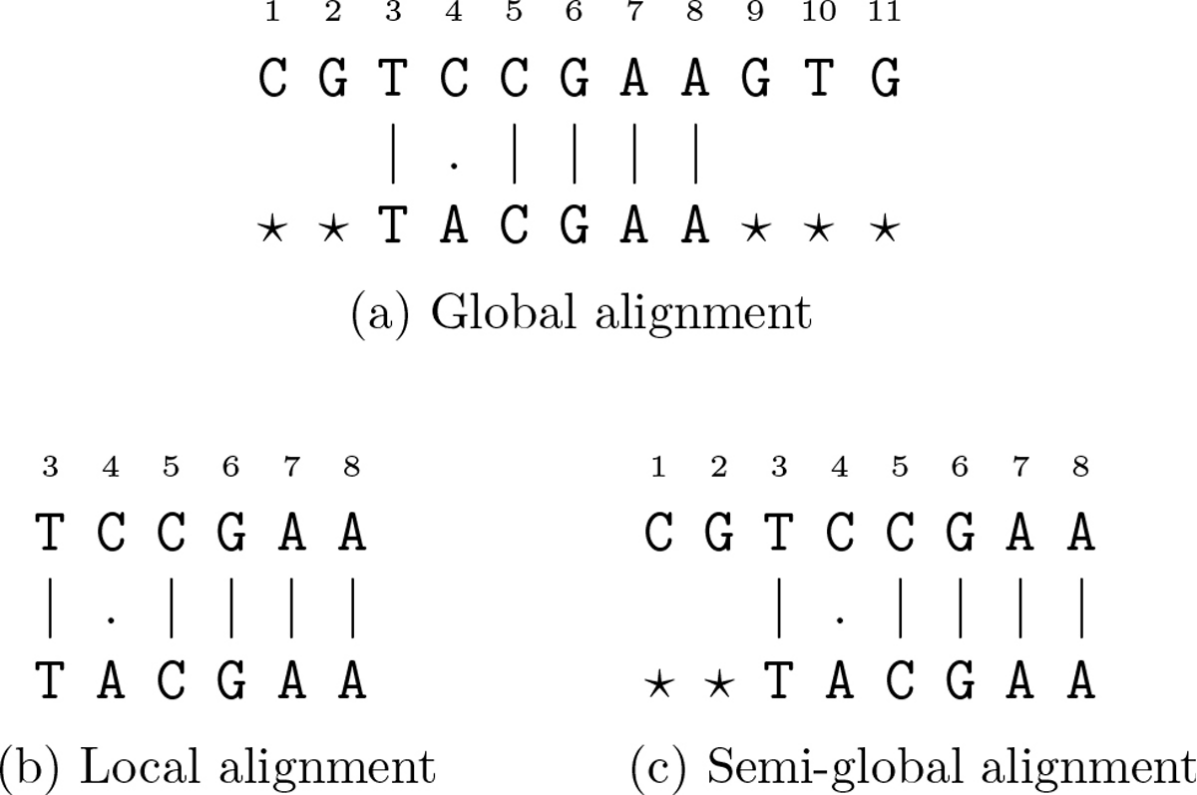
**Global, local, and semi-global alignment**. The global, local, and semi-global alignments between *t *= CGTCCGAAGTG and *x *= TACGAA. This figure was taken from [21].

**Example 1 (**[21]**) ***Let t *= ***CGTCCGAAGT **and x *= ***TACGAA**. Figures *2a, b, *and *2c* illustrate the global, the local, and the semi-global alignment, respectively*.

**Figure 3:**
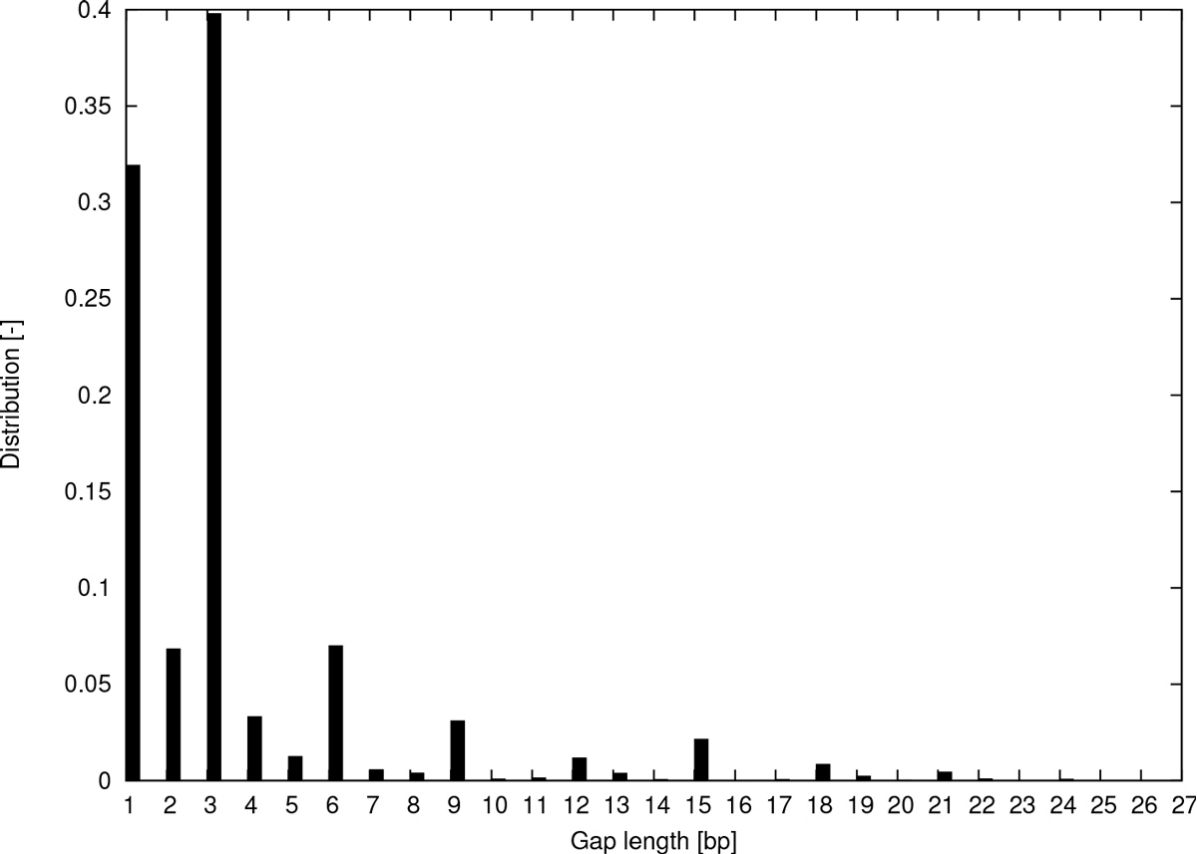
**Distribution of gap lengths in exome sequencing**. The distribution of gap lengths in exome sequencing. The data were generated by the Exome Sequencing Programme at the NIHR Biomedical Research Centre at Guy's and St Thomas' NHS Foundation Trust in partnership with King's College London. This figure was taken from [21].

Although gaps may occur in range of lengths, the short length of reads means large gaps can not be confidently detected directly. In Figure 3, the distribution of lengths of gaps in *homo sapiens *exome sequencing is demonstrated. The illustrated distribution agrees with the distribution in other studies on gaps (cf. [9,22,23]). Figure 3 represents a gap occurrence frequency of approximately 5.7 × 10**^-^**^6^ across the *exome*. This frequency is analogous to the ones observed in other studies on exome sequencing, plant genomes, and viruses (cf. [9,23,24]).

Moreover, Figure 3 shows an exponential decrease in the occurrence of gaps as the length increases and a preference for lengths which are multiples of 3. The presence of many gaps in short reads in the order of 25-150 base pairs (bp) is rather unlikely due to the low gap occurrence frequency. Hence, applying a traditional dynamic-programming approach, which essentially cannot bound the number of deletions and insertions in the alignment, would greatly affect the mapping confidence.

Motivated by the aforementioned observations, in [7], the authors presented GapMis, a tool for pairwise global and semi-global sequence alignment with a *single *gap. In this article, we present libgapmis, the analogous library implementation. libgapmis also includes two highly optimised versions: one based on Streaming SIMD Extensions (SSE); and one based on Graphics Processing Units (GPU). Proof of concept versions of GapMis and libgapmis were presented in [25] and [21], respectively. Millions of pairwise sequence alignments, performed here under realistic conditions based on the properties of real full-length genomes, demonstrate that libgapmis can increase the accuracy of extending short-read alignments end-to-end compared to more traditional approaches. The importance of our contribution is underlined by the fact that the provided open-source library functions can directly be integrated into any short-read alignment programme.

## Definitions and notation

In this section, we give a few definitions, generally following [26] and [7].

An *alphabet *∑ is a finite non-empty set whose elements are called *letters*. A *string *on an alphabet ∑ is a finite, possibly empty, sequence of elements of ∑. The zero-letter sequence is called the *empty string*, and is denoted by *ε*. The set of all the strings on the alphabet ∑ is denoted by ∑*. The *length *of a string *x *is defined as the length of the sequence associated with the string *x*, and is denoted by |*x*|. We denote by *x*[*i*], for all 1 ≤ *i *≤ |*x*|, the letter at index *i *of *x*. Each index *i*, for all 1 ≤ *i *≤ |*x*|, is a position in *x *when *x *≠ *ε*. It follows that the *i*th letter of *x *is the letter at position *i *in *x*, and that *x *= *x*[1 .. |*x*|]. A string *x *is a *substring *of a string *y *if there exist two strings *u *and *v*, such that *y *= *uxv*. Let *x*, *y*, *u*, and *v *be strings, such that *y *= *uxv *holds. If *u *= *ε*, then *x *is a *prefix *of *y*. If *v *= *ε*, then *x *is a *suffix *of *y*.

Let *x *be a non-empty string and *y *be a string. We say that there exists an *occurrence *of *x *in *y*, or, more simply, that *x occurs in y*, when *x *is a substring of *y*. Every occurrence of *x *can be characterised by a position in *y*. Thus we say that *x *occurs at the *starting position i *in *y *when *y*[*i* .. *i *+ |*x*| **- **1] = *x*. It is sometimes more suitable to consider the *ending position i *+ |*x*| **- **1. The *Hamming distance δ_H _*for two strings of the same length, is defined as the number of positions where the two strings have different letters. A *don't care *letter is a special letter that does not belong to alphabet ∑, and matches with itself as well as with any letter of ∑. It is denoted by ★. A *gap *is a finite sequence of such *don't care *letters. A *gap string *is a finite, possibly empty, sequence of elements of the alphabet ∑ ∪ **{★}**. Two letters *a *and *b *of alphabet ∑ **∪ {**★**} **are said to *correspond *(denoted by *a ***≈ ***b*) if they are equal, or, if at least one of them is the don't care letter. The *G-distance*, denoted by *δ_G_*, for two gap strings of the same length is defined as the number of positions in which the two strings possess letters that do not correspond. A gap string *x *is called *single-gap string *if there exist two strings *u *and *v *on alphabet ∑ and a gap *g*, such that *x *= *ugv*. Let conc(*y'*) be an operation that, given a gap string

$$y^{\prime }=y_{0}g_{0}y_{1}g_{1}\ldots {y_{n}}_{-2}g_{n-2}y_{n-1}$$

where *y_i _*∈ ∑*, for all 0 ≤ *i < n*, and *g_j _*∈ {★}*, for all 0 ≤ *j < n *- 1, returns the string *y *= *y*_0_*y*_1 _... *y*_*n*-1_, such that *y *∈ ∑*.

The *approximate string matching with k-mismatches and a single gap *problem can now be formally defined:

**Problem 1 (**[21]**) ***Given a text t of length n, a pattern x of length m *≤ *n, an integer k, such that *0 ≤ *k < m, and integers α and β, such that *0 ≤ *α *≤ *β and β < n, find all prefixes of t, such that for each prefix y*

• either *there exists a single-gap string y', with a gap g, such that y *= *conc*(*y'*), *δ_G_*(*x, y'*) ≤ *k, and α *≤ |*g*| ≤ *β;*

• or *there exists a single-gap string x', with a gap g, such that x *= *conc*(*x'*), *δ_G_*(*x', y*) ≤ *k, and α *≤ |*g*| ≤ *β;*

• or *δ_H _*(*x, y*) ≤ *k and α *= 0.

**Example 2 (**[21]**) ***Let t *= ***AGCAGAGGAGCAGGCGTTCCGTGGT**, x *= ***ACCGT**, k *= 2, *α *= 6, *and β *= 7. *A solution to this problem instance is the ending position *11, *since there exists a single-gap string x*' = ***ACC***★★★★★★***GT**, with a gap g *= ★★★★★★, *such that x *= ***conc***(**ACC**★★★★★★***GT***), *δ_G_*(*x*', *t*[1 .. 11]) = 2, *and *|*g*| = 6.

Let G[0 .. *n*, 0 .. *m*] be a matrix, where G[*i, j*] contains the minimum number of mismatches of the alignment between substring *t*[1 .. *i*] of *t *and substring *x*[1 .. *j*] of *x *allowing the insertion of a single gap either in *t*[1 .. *i*] or in *x*[1 .. *j*], for all 1 ≤ *i *≤ *n*, 1 ≤ *j *≤ *m*. For all 1 ≤ *j *≤ *m *and 1 ≤ *i *≤ *n*, we say that *x*[1 .. *j*] matches *t*[1 .. *i*] with at most *k*-mismatches and a single gap *if and only if *G[*i, j*] ≤ *k*. Matrix G is defined by the following recurrence [7].

$$\text{G}\left[i,j\right]=\left\{\begin{array}{cc} \text{min}\left\{\text{G}\left[i-1,j-1\right]+\delta_{H}\left(t\left[i\right],x\left[j\right]\right),\text{G}\left[j,j\right]\right\} & i>j\\ \text{min}\left\{\text{G}\left[i-1,j-1\right]+\delta_{H}\left(t\left[i\right],x\left[j\right]\right),\text{G}\left[i,i\right]\right\} & i<j\\ \text{G}\left[i-1,j-1\right]+\delta_{H}\left(t\left[i\right],x\left[j\right]\right) & i=j. \end{array}\right)$$

In order to compute the exact location of the inserted gap, either in the text or in the pattern, we also need to define a matrix H[0 .. *n*, 0 .. *m*] [7], such that

$$\text{H}\left[i,j\right]=\left\{\begin{array}{cc} j-i & \;\text{s}\text{.t}\;\text{G}\left[i,j\right]=\text{G}\left[i,i\right]\;\text{and}\;i<j\\ i-j & \;\text{s}\text{.t}\;\text{G}\left[i,j\right]=\text{G}\left[j,j\right]\;\text{and}\;i>j\\ 0 & \text{otherwise} \end{array}\right)$$

**Figure 4:**
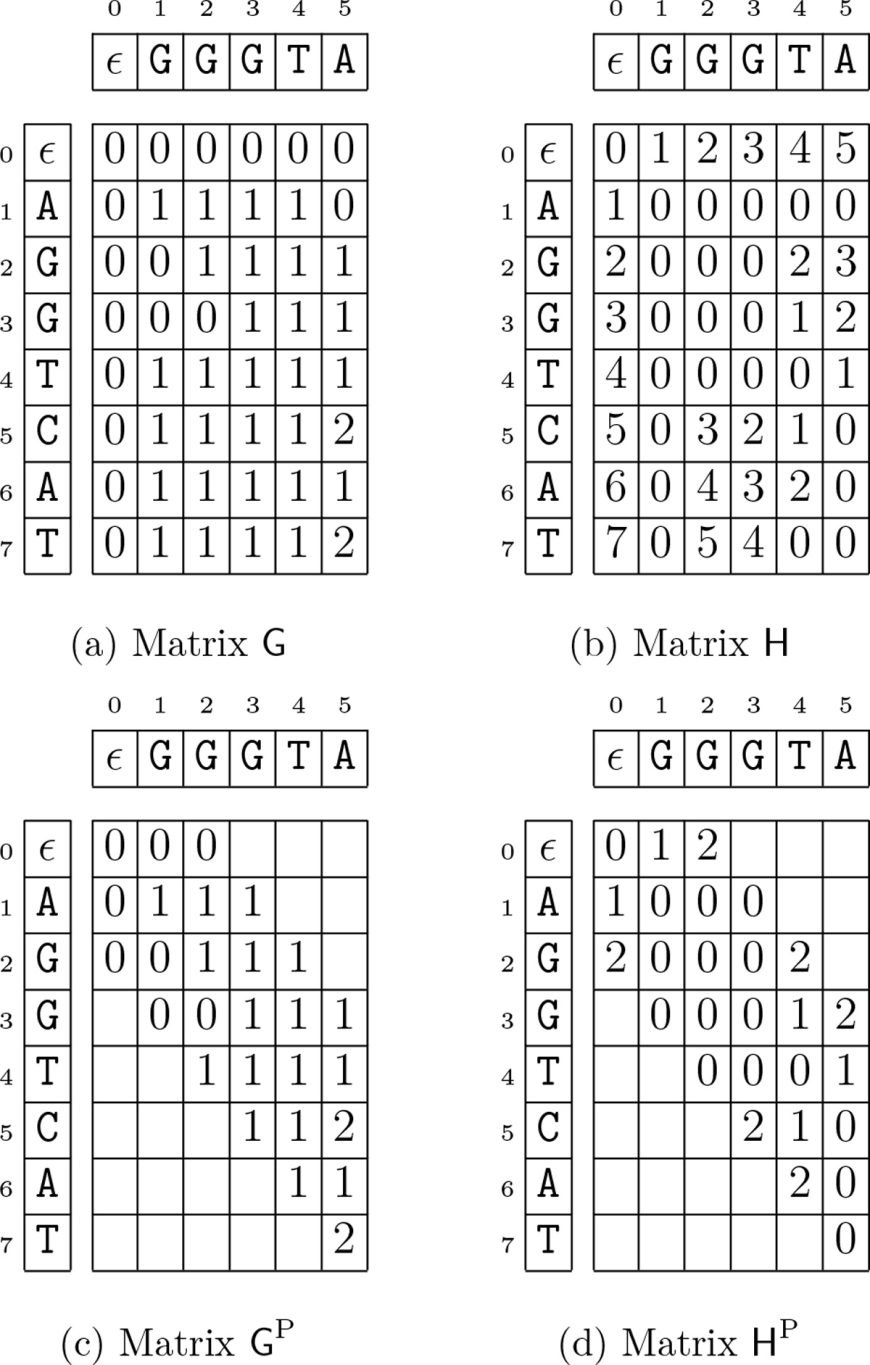
**Dynamic-programming matrices**. The matrices G, H, G^P^, and H^P ^for *t *= AGGTCAT, *x *= GGGTA, and *β *= 2. This figure was taken from [21].

**Example 3 (**[21]**) ***Let t *= ***AGGTCAT**, x *= ***GGGTA**, and β *= 2. Figure 4a and 4b* illustrate matrix G and matrix H, respectively*.

## Algorithm GapMis

Given the text *t *of length *n*, the pattern *x *of length *m*, and the threshold *β *as input, algorithm GAPMIS, first introduced in [7] (see Additional File 1), computes matrices G and H. In fact, we only need to compute a diagonal stripe (a narrow band) of width 2*β *+ 1 in matrix G and in matrix H. As a result, algorithm GAPMIS computes a pruned version of matrices G and H, denoted by G^P ^and H^P^, respectively (see Figure 4c and 4d).

**Proposition 1 (**[7]**) ***There exist at most *2*β *+ 1 *cells of matrix G that solve Problem 1*.

**Proposition 2 (**[7]**) ***Problem 1 can be solved by algorithm *GAPMIS* in time *O(mβ).

**Figure 5:**
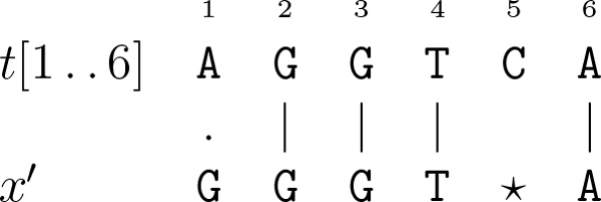
**Single-gap alignment**. The single-gap alignment between *t *= AGGTCAT and *x *= GGGTA for *k *= 1, *α *= 1, and *β *= 1. This figure was taken from [21].

**Example 4 (**[21]**) ***Let t *= ***AGGTCAT***, *x *= ***GGGTA***, *k *= 1, *a *= 1, *and β *= 1. *Starting the trace-back from cell H*[6, 5] *(see *Figure 4d*), yields a solution since G*[6, 5] ≤ 1 *(see *Figure 4c*). Trivially, the inserted gap is in the pattern, and its length is *1. *Finally, we can find the position of the inserted gap (position 5) using matrix H. Hence, a solution to this problem instance is the ending position *6 *(see *Figure 5*)*, *since there exists a single-gap string x*' = ***GGGT***★***A***, *with a gap g *= ★, *such that x *= ***conc***(***GGGT***★*A*), *δ_G_*(*x'*, *t*[1 .. 6]) = 1, *and *|*g*| = 1.

Alternatively, we could compute matrix G and matrix H based on a simple *alignment score *scheme depending on the application of the algorithm (see the following section or [27], for example), and compute the *maximum score *in time Θ(*β*) by Proposition 1.

## Library libgapmis

In this section, we give a brief description of the library implementation. libgapmis was implemented in the C programming language. First, we start by describing the standard CPU version of the library. Thereafter, we discuss some technical issues regarding the SSE- and GPU-based implementations. Finally, we describe how the provided functions are extended to accommodate a variable, but bounded, number of gaps in the alignment.

Algorithm GAPMIS was implemented as a function computing matrices G and H based on a simple alignment score scheme. The scheme uses the scoring matrix EDNAFULL [28] (resp. EBLOSUM62 [29]) for DNA (resp. protein) sequences to assign scores for every possible nucleotide (resp. residue) match or mismatch. Moreover, it uses affine gap penalty to score the insertion of a gap of *n >*0 positions as follows:

gapopeningpenalty+(n-1)×gapextensionpenalty.

Finally, the total score for each alignment is obtained by adding these two scores: scoring matrix and affine gap penalty scores. The optimal alignment is the alignment with the highest such total score. The same alignment score scheme is applied in package EMBOSS [30].

We implemented the following functions:

• gapmis_one_to_one: this function finds the optimal semi-global alignment between two sequences. It first implements algorithm GAPMIS in time O(mβ); thereafter, it finds the optimal semi-global alignment in time O(β). Finally, gapmis_one_to_one finds the position of the single gap via backtracking in matrix H in time O(m). The user can omit computing the position of the single gap and thereby computing matrix H.

• gapmis_one_to_many: this function uses function gapmis_one_to_one as building block. It computes the *ℓ *optimal semi-global alignments between a *query sequence *and a set of *ℓ target sequences*.

• gapmis_many_to_many: this function uses function gapmis_one_to_many as building block. It computes the *κ *× *ℓ *optimal semi-global alignments between a set of *κ *query sequences and a set of *ℓ *target sequences.

Finally, we implemented functions results_one_to_one, results_one_to_many, and results_many_to_many for generating the visualisation of the analogous output in a format similar to the one generated by EMBOSS.

### SSE-based implementation

The SSE-based implementation is a direct application of the *inter-sequence vectorisation *scheme. It has been used to accelerate the Smith-Waterman algorithm and analogous dynamic-programming algorithms [31,32]. Algorithm GAPMIS, under this vectorisation scheme, uses SSE instructions to simultaneously compute multiple separate matrices (usually 2, 4, or 8 depending on the vector width and the data type used) corresponding to alignments of one query sequence against multiple other target sequences.

Currently, the vectorisation uses 32 bit floating-point arithmetics to represent scores, implying that, on CPUs with SSE3 vector units, a vector width *w *:= 4 is used. By restricting scores to integer values and using 16 bit integers, we may increase the vector width to *w *:= 8. For performance-related reasons, the SSE-based version only supports the computation of alignment scores, and, therefore, does not support backtracking. The functions provided are gapmis_one_to_many_opt_sse and gapmis_many_to_many_opt_sse, which make use of the aforementioned vectorisation scheme to compute the scores for each pair of sequences. Finally, we make use of the purely sequential function gapmis_one_to_one to find the position of the single gap via backtracking in matrix H. In order to further accelerate the computations, the user may optionally and transparently execute these functions on multi-core architectures by setting the number of threads. More technical details of the SSE-based implementation can be found in [21].

### GPU-based implementation

The function gapmis_one_to_one was ported to GPUs using OpenCL in order to maintain a vendor-independent GPU version. In analogy to the SSE-based implementation, only the computation of alignment scores are offloaded to the GPU. The GPU implementation is also similar to the SSE-based implementation in the sense that multiple dynamic-programming matrices are computed simultaneously.

**Figure 6:**
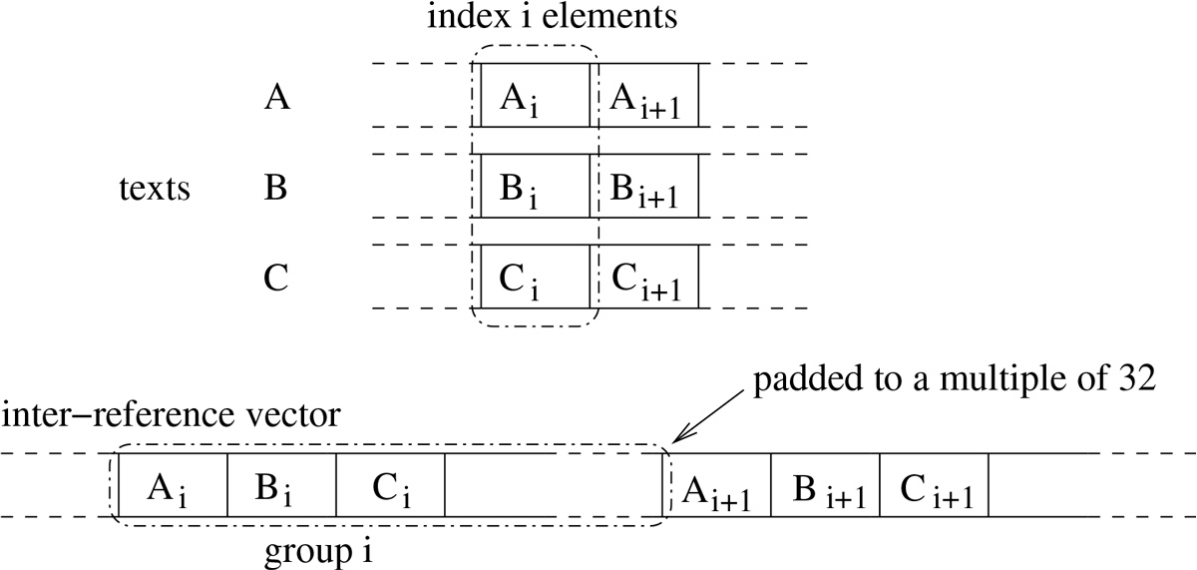
**Inter-sequence GPU memory organisation**. The inter-sequence GPU memory organisation. This figure was taken from [21].

Aligning a set of query sequences $\bar{x}=\left\{x_{1},\ldots ,x_{k}\right\}$ against a set of target sequences $\bar{t}=\left\{t_{1},\ldots ,t_{\ell }\right\}$ is achieved by launching a total of *κ *× *ℓ *threads on the GPU to exploit inter-sequence parallelism--similar to the aforementioned SSE vectorisation scheme. GPU threads are grouped such that every thread group aligns one query sequence against all target sequences. Each thread in a thread group computes a different dynamic-programming matrix sequentially and independently of all other threads. Due to the independence between the individual alignment tasks, we refer to this scheme as *inter-task parallelisation*. In order to prevent memory-access conflicts and also maximise memory throughput, an inter-sequence device memory organisation scheme is applied (see Figure 6 in this regard).

Similar to the SSE-based version, the functions provided are gapmis_one_to_many_opt_gpu and gapmis_many_to_many_opt_gpu. Finally, we make use of the purely sequential function gapmis_one_to_one to find the position of the single gap via backtracking in matrix H. More technical details of the GPU-based implementation can be found in [21].

### Accommodating multiple gaps

The presence of multiple gaps is unlikely given the observed gap occurrence frequency in real-life applications: 5.7 × 10^-6 ^in the human exome (see the Background Section), 1.7 × 10^-5 ^in *Beta vulgaris *[24], 2.4 × 10^-5 ^in *Arabidopsis thaliana *[24], and 3.2 × 10^-6 ^in *bacteriophage PhiX174 *[24]. However, in order to increase the flexibility of our library, we implemented two additional functions, gapmis_one_to_one_f and gapmis_one_to_one_onf, to allow for a variable, but bounded, number of gaps in the alignment.

• gapmis_one_to_one_f: this function provides the user the option to split the query sequence into *f *fragments, based on the observed gap occurrence frequency and the query length, by taking the number of fragments as input argument. It then uses function gapmis_one_to_one to perform a single-gap alignment for each fragment independently. The total score of the alignment is obtained by adding the *f *individual scores of the fragments. We denote this function by gm -f <int>, where <int> is the number of fragments *f *used as input argument.

• gapmis_one_to_one_onf: this function computes the alignment by using the optimal number of fragments. First, it takes the maximum number of fragments as input argument, say *f*_max_, and only computes the total score of the alignments, for each different number 1, 2, ..., *f*_max _of fragments. It then uses function gapmis_one_to_one_f to compute the alignment by passing the optimal number of fragments--the one that gives the maximum total score in the previous step--as input argument. We denote this function by gm -onf <int>, where <int> is the maximum number of fragments *f*_max _used as input argument.

## Experimental results

The experiments were conducted on a Desktop PC using up to 4 cores of Intel i7 2600 CPU at 3.4 GHz under Linux, and an NVIDIA GeForce 560 GPU with 336 CUDA cores and 1 GB DDR5 device memory. libgapmis is distributed under the GNU General Public License (GPL). The library is available at http://www.exelixis-lab.org/gapmis, which is set up for maintaining the source code and the man page documentation.

To the best of our knowledge, lipgapmis is the first library for extending pairwise short-read alignments. The main design goal of lipgapmis is to identify a single gap in the alignment (see the Background Section for the motivation). Therefore, in this section, we focus on comparing the performance of function gapmis_one_to_one to the analogous performance of EMBOSS needle. The latter implements Needleman-Wunsch algorithm for semi-global alignment. The Needleman-Wunsch algorithm is the traditional approach used for semi-global alignment. needle is, up-to-date, one of the most popular pairwise sequence alignment programmes for global and semi-global alignment.

We generated 100, 000 pairs of 100 bp-long sequences on the DNA alphabet. Initially, each pair consisted of two identical sequences. Subsequently, we inserted:

• *a single gap *with a uniformly random length that ranged between 1 and 30 into one of the two sequences;

• a uniformly random number of *mismatches *that ranged between 1 and 10.

Since the presence of multiple gaps is unlikely based on the gap occurrence frequency observed in real datasets, this experimental setting aims to demonstrate the suitability of the proposed algorithm compared to more traditional approaches in identifying the simulated inserted gap.

We seamlessly integrated function gapmis_one_to_one into a test programme, denoted by gapmis, for computing the optimal semi-global alignment between a pair of sequences. In each case, for a fair comparison of needle and gapmis, an effort was made to run the programmes under as similar conditions as possible. In gapmis, we additionally used function results_one_to_one to produce the corresponding output. While parsing the output generated by the two programmes, we considered any inserted gap as gap, excluding, however, a gap inserted in the end of the alignment.

We consider as *valid *those alignments where the number of inserted gaps is less or equal to the ones originally inserted. Furthermore, we consider as *correct *those valid alignments with gaps whose total length is smaller or equal to the length of the ones originally inserted *and *with number of mismatches being less or equal to the ones originally inserted.

**Figure 7:**
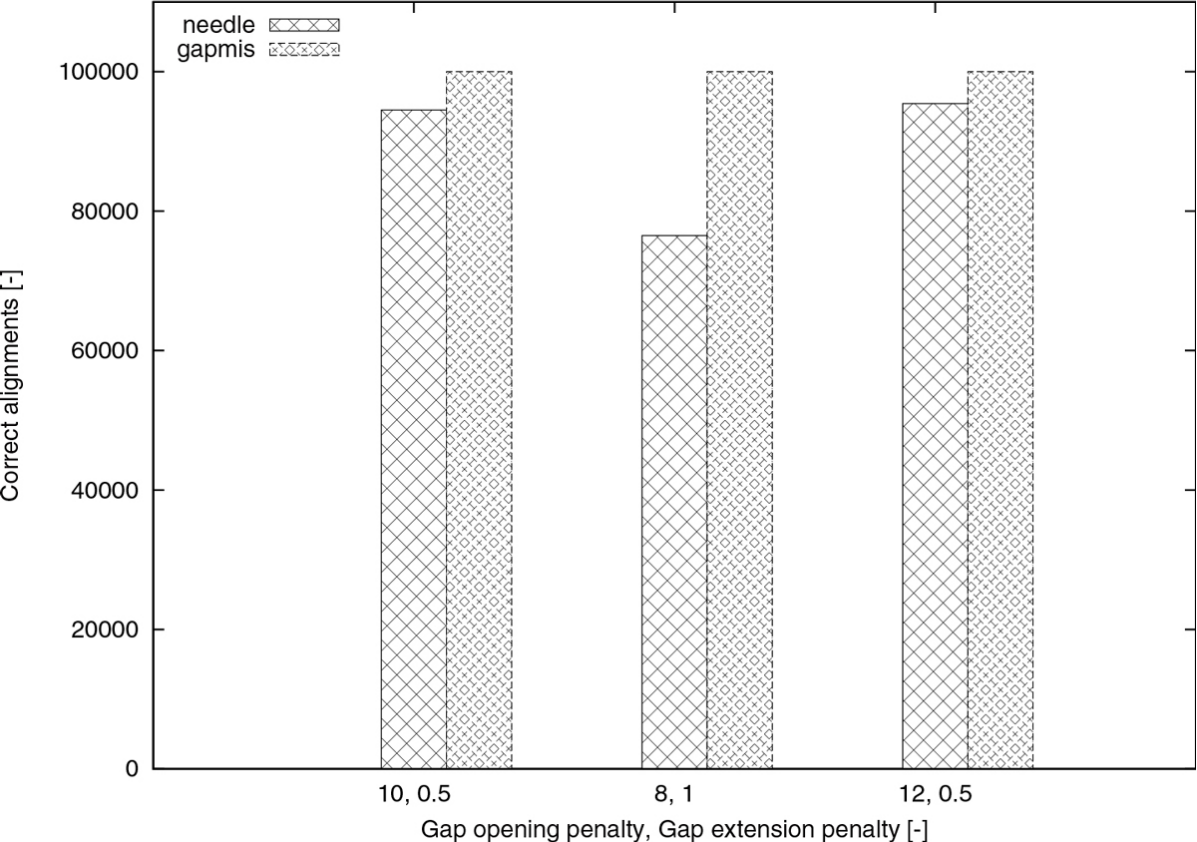
**Correct alignments**. The correct alignments of Tables 1-3.

The above experimental procedure was repeated using different gap opening and gap extension penalties. As corroborated by the results in Tables 1, 2, 3, gapmis is more suitable for identifying single alignment gaps in all cases. As it is also shown in [7] and [21], needle cannot--by design--guarantee the insertion of at most one gap into the alignment, even when setting the gap opening penalty to 12.0 and the gap extension penalty to 0.5. The correct (as per our definition) alignments of Tables 1, 2, 3 are illustrated in Figure 7. Furthermore, we compared the processing times of gapmis to those of needle by generating 10,000 pairs of 100, 150, 200, and 250 bp-long DNA sequences in analogy to the aforementioned experiment. We used two different versions of gapmis: one with the modifier -m 30 to set *β *= 30; and one with *β *= *n *- 1, where *β *is the maximum allowed length of the single gap, and *n *is the length of the longest sequence.

**Table 1:** Valid and correct alignments with gap opening penalty 10.0 and gap extension penalty 0.5.

Programme	Valid	Correct
Needle	94,552	94,516
Gapmis	100,000	99,996

The valid and correct alignments of 100, 000 pairs of 100 bp-long generated sequences with gap opening penalty 10.0 and gap extension penalty 0.5.

**Table 2:** Valid and correct alignments with gap opening penalty 8.0 and gap extension penalty 1.0.

Programme	Valid	Correct
needle	76,512	76,501
gapmis	100,000	99,997

The valid and correct alignments of 100, 000 pairs of 100 bp-long generated sequences with gap opening penalty 8.0 and gap extension penalty 1.0.

**Table 3:** Valid and correct alignments with gap opening penalty 12.0 and gap extension penalty 0.5.

Programme	Valid	Correct
needle	95,452	95,427
gapmis	100,000	99,999

The valid and correct alignments of 100, 000 pairs of 100 bp-long generated sequences with gap opening penalty 12.0 and gap extension penalty 0.5.

**Figure 8:**
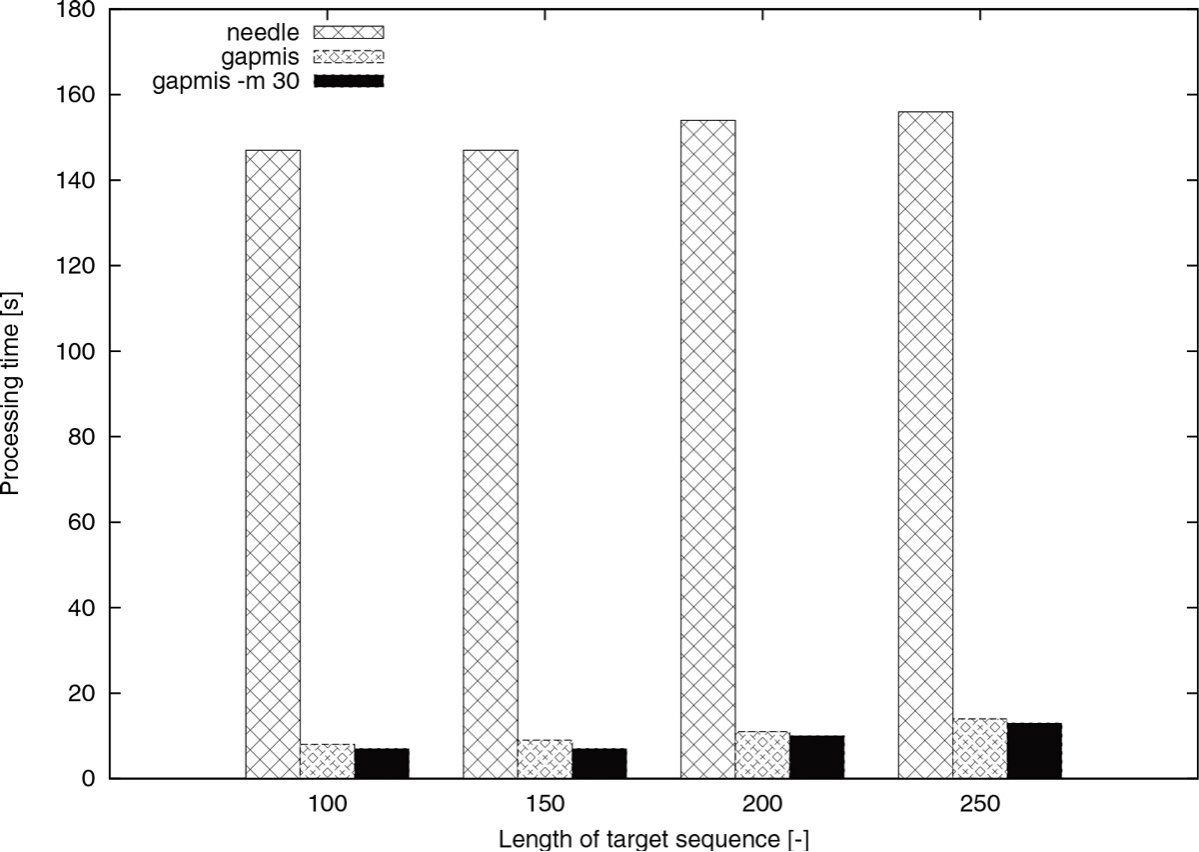
**Processing times of ****needle**** and ****gapmis**. The processing times of needle and gapmis for aligning 10, 000 pairs of sequences.

The results in Figure 8 show that gapmis was able to complete the assignment up to 20× faster than needle. Although the asymptotic complexity of the two algorithms is the same, the number of arithmetic operations required by algorithm GAPMIS is substantially lower. This can be easily explained by examining the recurrence relations of the two algorithms. The version with the modifier -m 30 was always the fastest confirming our theoretical results. Note that, it only computes a narrow band in the dynamic-programming matrices (see Figure 4c and 4d).

We also evaluated the time efficiency of the accelerated SSE- and GPU-based versions of libgapmis, by comparing their processing times against the ones of the standard CPU version. In particular, we generated a 75 bp-long DNA query sequence and 4, 639, 576 100 bp-long DNA target sequences. This represents a realistic setting for re-sequencing applications because the *seed *part of a short read usually occurs in thousands or millions of positions along the reference sequence. Hence an important problem in re-sequencing is the efficient and accurate *extension *of these thousands to millions of potential alignments. We used the following versions of the function gapmis_one_to_many:

• the CPU version;

• the single-core SSE version;

• the SSE version with 4 threads (-t 4);

• the GPU version.

**Figure 9:**
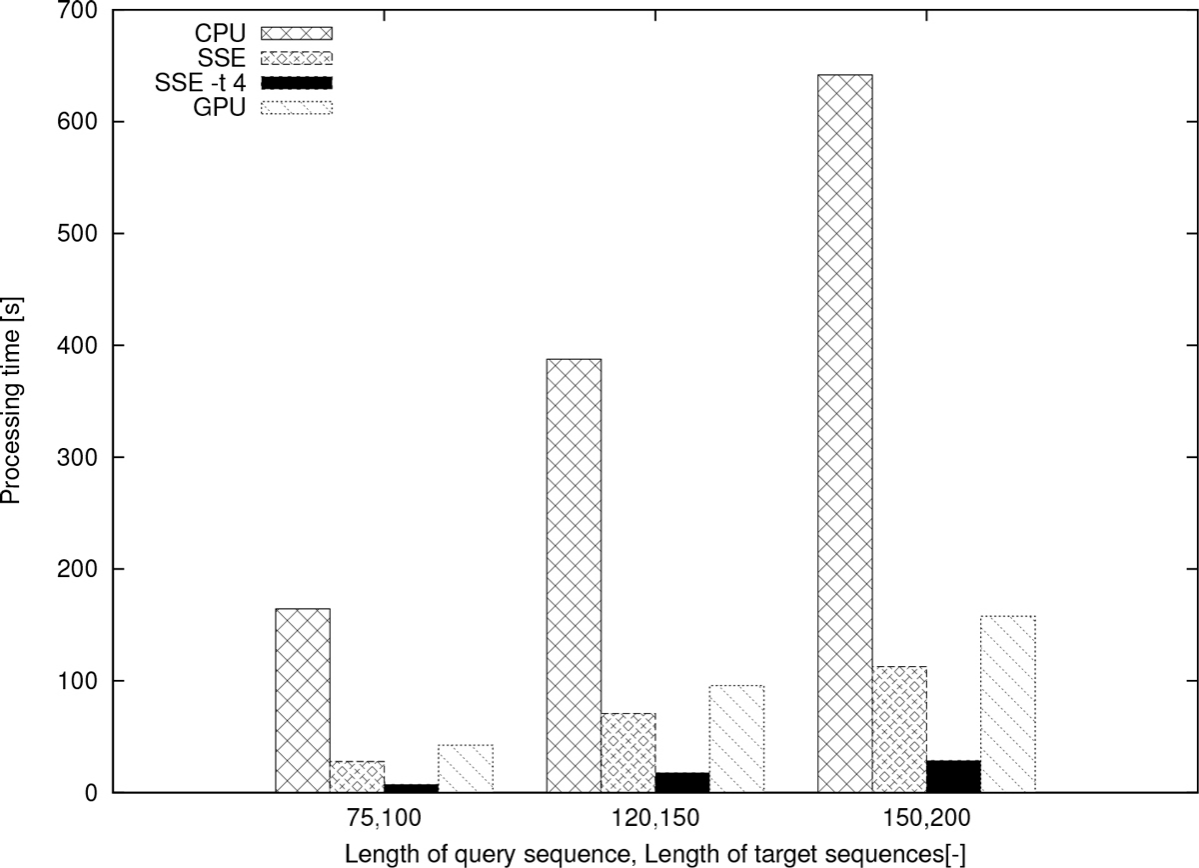
**Processing times of ****gapmis_one_to_many**. The processing times of gapmis_one_to_many for aligning a query sequence and 4, 639, 576 target sequences.

The same experiment was repeated with 150 and 200 bp-long sequences. As shown by the results in Figure 9, the single-core SSE version accelerates the computations by a factor of 6 compared to the CPU version; the SSE -t 4 version by a factor of 23 compared to the CPU version; and the GPU version by a factor of 4 compared to the CPU version. The cell updates per second (CUP/s) are 290 MCUP/s, 1.6 GCUP/s, 6.5 GCUP/s, and 1.2 GCUP/s, for the CPU, the SSE, the SSE -t 4, and the GPU versions, respectively.

**Figure 10:**
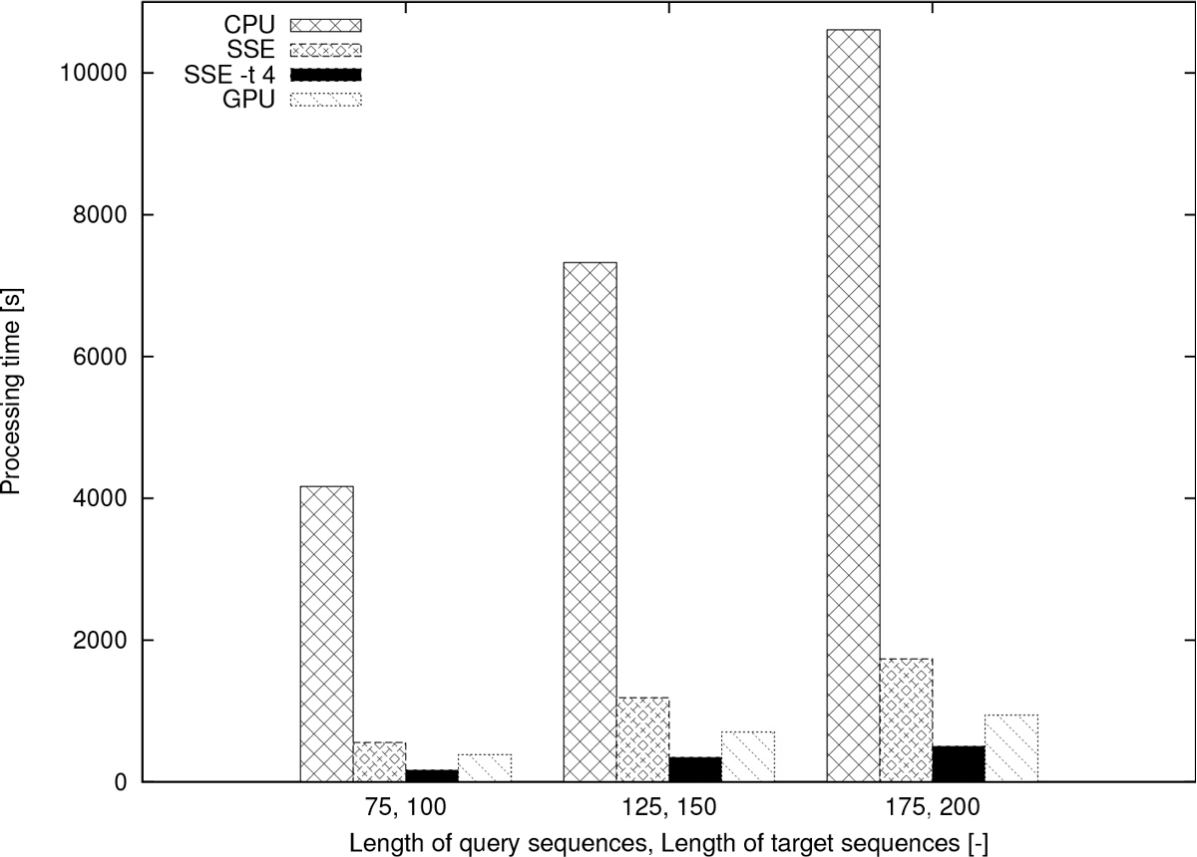
**Processing times of ****gapmis_many_to_many**. The processing times of gapmis_many_to_many for aligning 1, 000, 000 query sequences and 200 target sequences.

As further experiment, we generated 1, 000, 000 75 bp-long DNA query sequences and 200 100 bp-long DNA target sequences. Similar to the above experiment, the four aforementioned versions of function gapmis_many_to_many were used, and the same experimental procedure was repeated with 150 and 200 bp-long sequences. As shown by the results in Figure 10, the single-core SSE version accelerates the computations by a factor of 6 compared to the CPU version; the SSE -t 4 version by a factor of 20 compared to the CPU version; and the GPU version by a factor of 11 compared to the CPU version. The CUP/s are 190 MCUP/s, 1.1 GCUP/s, 4 GCUP/s, and 2.2 GCUP/s, for the CPU, the SSE, the SSE -t 4, and the GPU versions, respectively.

As further experiment, in order to evaluate the performance of programme gapmis, function gapmis_one_to_one_f, function gapmis_one_to_one_onf, and needle, under real conditions, we simulated 1, 000, 000 100 bp-long query sequences from the 30 Mbp chromosome 1 of *Arabidopsis thaliana *(AT) obtained from [33], and inserted mismatches and gaps into the reference sequence; then we aligned them back against the original reference sequence. As mismatch occurrence frequency and gap occurrence frequency we used 1.6 × 10**^-^**^3^and 2.4 × 10**^-^**^5^, respectively--the ones observed in AT [24]. Since, in practice, insertions occur less frequently than deletions, 42% of the inserted gaps were insertions and 58% deletions--also observed in AT [24]. For the length of the inserted gaps, we used the distribution of gap lengths shown in Figure 3, which is consistent with other studies on gap distributions (cf. [9,22,23]). Since the queries were simulated, we were able to know the exact location of the fragments of the reference sequence they were derived from (the target sequences). Hence, we were able to classify each generated alignment as valid/invalid and correct/incorrect. We define *accuracy *as the proportion of correct alignments in the dataset. Thus, we evaluated the accuracy of the aforementioned programmes in *extending *an alignment end-to-end, assuming that the *seed *part of the alignment is already performed by using a conventional indexing scheme, that is, a hash-based index [15] or an FM index [16]. We repeated the same experiment by simulating 150 bp-long query sequences and using other gap occurrence frequencies--observed in *Beta vulgaris *(BV) [24] and *Homo sapiens *(HS) exome [9].

The high accuracy of libgapmis is demonstrated by the results shown in Table 4. The results show that function gm -onf 3 has the highest accuracy in all cases. It can increase the accuracy of extending short-read alignments end-to-end by 0.01% compared to needle. Given the observed gap occurrence frequencies, the increased accuracy of gap identification is significant. For instance, the proportion of pairs of sequences with gaps in the six datasets of Table 4 ranged from 0.85% to 3.5%.

**Table 4:** Correct alignments using gapmis, gapmis_one_to_one_f, gapmis_one_to_one_onf, and needle.

Species	Length of queries [bp]	Gap occurrence frequency	**gapmis**	**gm -f 2**	**gm -f 3**	**gm -onf 2**	**gm -onf 3**	**needle**
AT	100	2.4 **× **10**^-^**^5^	999,099	998,404	997,561	999,207	**999,259**	999,126
AT	150	2.4 **× **10**^-^**^5^	998,805	998,171	997,542	999,024	**999,152**	999,115
BV	100	1.7 **× **10**^-^**^5^	999,361	998,868	998,229	999,432	**999,459**	999,353
BV	150	1.7 **× **10**^-^**^5^	999,196	998,771	998,249	999,347	**999,432**	999,378
HS	100	5.7 **× **10**^-^**^6^	999,809	999,615	999,419	999,822	**999,825**	999,782
HS	150	5.7 **× **10**^-^**^6^	999,795	999,606	999,408	999,817	**999,825**	999,793

The correct alignments of 1, 000, 000 pairs of simulated sequences with various observed gap occurrence frequencies using gapmis, gapmis_one_to_one_f, gapmis_one_to_one_onf, and needle. Each of the datasets consists of 1,000,000 pairs of sequences; the highest number of correct alignments for each dataset is shown in bold.

Although the gap opening penalty in needle could be increased by the user, this would have a potentially fatal impact on accuracy because the high number of mismatches opted would be underestimated [21]. We checked this assumption by conducting the following last experiment. We obtained 100, 000 100 bp-long and 100, 000 150 bp-long query sequences from the 30 Mbp chromosome 1 of AT, and inserted mismatches and gaps into the reference sequence; then we aligned them back against the original reference sequence using needle, similar to the previous experiments. The gap opening penalty ranged from 10.0 to 20.0, and the gap extension penalty was set to 0.5. Our assumption is confirmed by the results shown in Table 5. Notice that, increasing the gap opening penalty increases the valid alignments but has a negative impact on the accuracy of needle: the number of correct alignments decreases.

**Table 5:** Valid and correct alignments using needle.

Programme	Species	Length of queries [bp]	Gap occurrence frequency	Gap opening penalty	Gap extension penalty	Valid alignments	Correct alignments
needle	AT	100	2.4 × 10^-5^	10.0	0.5	99,988	**99,917**
needle	AT	100	2.4 × 10^-5^	15.0	0.5	99,992	99,911
needle	AT	100	2.4 × 10^-5^	20.0	0.5	**99,996**	99,850
needle	AT	150	2.4 × 10^-5^	10.0	0.5	99,991	**99,919**
needle	AT	150	2.4 × 10^-5^	15.0	0.5	99,992	99,901
needle	AT	150	2.4 × 10^-5^	20.0	0.5	**99,996**	99,834

The valid and correct alignments of 100, 000 pairs of simulated sequences with the gap occurrence frequency observed in *Arabidopsis thaliana *using needle. Each of the datasets consists of 100,000 pairs of sequences; the highest numbers of correct and valid alignments for each dataset are shown in bold.

## Conclusions

In this article, we presented libgapmis, an ultrafast and flexible library for extending pairwise short-read alignments end-to-end. Apart from the standard CPU version, it includes ultrafast SSE- and GPU-based implementations. libgapmis is based on GapMis, a tool that computes a different version of the traditional dynamic-programming matrix for sequence alignment.

This work is directly motivated by the next-generation re-sequencing application. We demonstrated that libgapmis is more suitable and efficient than more traditional approaches for extending short-read alignments end-to-end. Adding the flexibility of bounding the number of gaps inserted in the alignment, strengthens the classical scheme of scoring matrices and affine gap penalty scores. The presented experimental results are very promising, both in terms of identifying gaps and efficiency.

By exploiting the potential of modern CPU and GPU architectures and applying multi-threading, we improved the performance of the purely sequential CPU version by more than one order of magnitude. More importantly, the functions provided in libgapmis can be directly integrated into any short-read alignment programme. Our immediate target is to further optimise the code, and also integrate the functions of this library into a short-read alignment pipeline.

## Competing interests

The authors declare that they have no competing interests.

## Authors' contributions

SPP and AS designed the study. NA, SB, TF, and SPP developed the library. TF and SPP conducted the experiments. SPP wrote the manuscript with the contribution of all authors. The final version of the manuscript is approved by all authors.

## Supplementary Material

Additional file 1 **Algorithm GAPMIS**. The algorithm GAPMIS computes matrices G and H. It takes as input the text *t *of length *n*, the pattern *x *of length *m*, and the threshold *β*. This algorithm was taken from [7]. Click here for file
