# Supplementary material for: libgapmis: extending short-read alignments
Source: BMC Bioinformatics. 2013 Nov 4;14(Suppl 11):S4. doi: 10.1186/1471-2105-14-S11-S4 (PMC3821552; doi:10.1186/1471-2105-14-S11-S4)
Supplement: Additional file 1 — Algorithm GAPMIS. The algorithm GAPMIS computes matrices G and H. It takes as input the text t of length n, the pattern x of length m, and the threshold β. This algorithm was taken from [7]. [file 1471-2105-14-S11-S4-S1.pdf]

ALGORITHM GapMis(t, n, x, m, β)

```
{Initialise matrices G and H }
for i ← 0 to n do
    G[i, 0] ← 0;
    H[i, 0] ← i;
for j ← 0 to m do
    G[0, j] ← 0;
    H[0, j] ← j;
{Computing matrices G and H}
for i ← 1 to min{n, m + β} do
    for j ← max{1, i − β} to min{m, i + β} do
        if i < j then
            u ← G[i − 1, j − 1] + δ_H(t[i], x[j]);
            v ← G[i, i];
            G[i, j] ← min{u, v};
            if v < u then
                H[i, j] ← j − i;
            else
                H[i, j] ← 0;
        if i > j then
            u ← G[i − 1, j − 1] + δ_H(t[i], x[j]);
            v ← G[j, j];
            G[i, j] ← min{u, v};
            if v < u then
                H[i, j] ← i − j;
            else
                H[i, j] ← 0;
        if i = j then
            G[i, j] ← G[i − 1, j − 1] + δ_H(t[i], x[j]);
            H[i, j] ← 0;
return G and H;
```
